# Characterization of notifications of violence against women living in rural contexts in Brazil from 2011 to 2020

**DOI:** 10.1590/1980-549720240059

**Published:** 2024-12-13

**Authors:** Luciane Stochero, Liana Wernersbach Pinto

**Affiliations:** I Fundação Oswaldo Cruz, Escola Nacional de Saúde Pública Sergio Arouca – Rio de Janeiro (RJ), Brazil.

**Keywords:** Violence against women, Gender-based violence, Rural population, Health information systems, Notification

## Abstract

**Objective:**

To describe and analyze notifications and the temporal trend of violence against women living in rural contexts in Brazil, from 2011 to 2020.

**Methods:**

Ecological time-series study of a descriptive and analytical nature, with data from the Notifiable Diseases Information System on violence against women aged 18 to 59 years, in rural areas, from 2011 to 2020, in Brazil. The analyses were descriptive and trend-related, with the regression model using inflection points (joinpoint) and calculation of the annual percent change (APC) and the average annual percent change (AAPC).

**Results:**

A total of 79,229 notifications of violence against rural women were recorded. The most reported violence was physical (77.6%), psychological/moral (36.5%), and sexual (6.2%). They occurred, above all, among young, Black, married women with low levels of education. They were committed, in most cases, at home and, mainly, by a male partner. APC was statistically increasing throughout the studied period in Brazil, in the North, Midwest, South, and Northeast regions, including 18 states and the Federal District (DF). As for AAPC, all country, region, state, and DF rates showed a statistically increasing trend.

**Conclusion:**

In view of the increasing reported cases of violence against rural women throughout the country, which are mainly committed by people they are related to and in their own home, it is encouraged to reinforce the training of health professionals to improve and expand the process of notification as an instrument of care for women in situations of violence.

## INTRODUCTION

Violence against women is understood as any action or conduct based on gender, including that resulting from discrimination or ethnic inequality, that causes death, harm, or physical, sexual, or psychological suffering^1^. In Brazil, it is estimated that, on average, 18.6 million women aged 16 and over suffered some form of violence throughout 2022; the most frequent were: verbal abuse, stalking, threats, and physical assault^2^.

The data, like most research, do not differentiate between women living in urban and rural areas, and this lack of information regarding women living in rural contexts contributed to the invisibility of the issue and, consequently, fewer actions to combat violence in this context^3^.

The rural context encompasses a series of particularities, such as the absence of neighbors and relatives, lack of financial autonomy, social and geographic isolation, and distance from health and protection services, which are normally located in urban centers^4–6^. Hence, "rural women find empowerment in adversities such as exclusion and difficulties in accessing health and safety services"^4^. These adversities, rooted in gender oppression, contribute to strengthening the silencing of these women.

Among the few publications on violence against women living in rural contexts, we highlight a study whose authors used data from the Notifiable Diseases Information System (*Sistema de Informação de Agravos de Notificação* – SINAN), from 2010 to 2012, with 7,197 notifications against adult Brazilian rural women. The most reported types of violence were physical (76.8%), psychological/moral (38.4%), and sexual (7.4%)^2^. Authors of another more recent study, with data from the National Survey of Health (2019), observed the experience of psychological (18.0%), physical (4.4%), and sexual (1.5%) violence, in the last 12 months, by women in rural areas aged 18 to 59 years^7^. In both studies, the aggressors were mostly acquaintances, and the residence was the main place of occurrence^7^.

The health sector has an important role in tackling violence against women^4,8^. Health professionals are responsible for identifying, welcoming, and attentively and carefully listening to women, guiding, monitoring, and referring cases of violence as well as recording them in medical records and reporting them^8^. Notification is a key element for women's comprehensive care, removing cases of violence from invisibility, preventing repeated violence, and allowing for the network of protection and guarantee of rights to be activated and articulated^9^.

Violence against rural women is a public health issue. Rural contexts can increase violence, prevent people from asking for help, and cause women to remain in situations of violence for longer. Nationwide studies on this topic are still scarce, making the problem invisible. In the present study we aim to help fill this gap and expand knowledge on this topic. Our objective is to describe and analyze the notifications and temporal trends of violence against women living in rural contexts in Brazil, from 2011 to 2020.

## METHODS

### Study design

This is an ecological, time-series study of a descriptive and analytical nature, using as a data source the SINAN notification forms on violence against women aged 18 to 59 years, living in rural areas, in the period from 2011 to 2020, from all states in Brazil. The choice of the beginning of the analysis period was due to Ordinance No. 104/2011, according to which domestic and sexual violence was defined as a compulsorily notifiable offense and notifications of violence for all health services were universalized^9^.

### Study variables

Types of violence: physical, psychological/moral, sexual, torture, financial/economic, self-inflicted, human trafficking, neglect/abandonment, violence by legal intervention, and others^10^.

In the "area of residence" field, the rural option was used, defined as "an area with characteristics typical of the countryside, with a dispersed population, relatively far from administrative centers, limited access to public services, agricultural production"^10^.

Sociodemographic variables: age (18–29, 30–39, 40–49, and 50–59 years), ethnicity/skin color (white, brown/Black, Indigenous, Asian, and unknown), marital status (single, married/consensual union, widow, separated, unknown), and level of education (illiterate/some elementary school, elementary school/some high school, high school/some college education, and college education).

Event characteristics variables: place of occurrence (residence/collective housing, the street, other locations — school, sports venue, bar/similar, shops/services, industries/construction, others —, unknown) and recurrence (yes, no, unknown).

Aggressor's characteristics variables: sex of the likely aggressor (man, woman, both sexes, unknown), number of people involved (one, two or more, unknown), and relationship with the likely aggressor (spouse/boyfriend, former spouse/ex-boyfriend, stranger, relative — father, mother, stepmother, stepfather, son, brother —, friends/acquaintances, others — person with institutional relationship, boss/employer, police officer/law enforcement officer).

Referral location variables: health network, social welfare network, office on violence against women, public prosecutor's office, women's police station, other police stations, human rights reference center, and public defender's office.

For the temporal analysis, the variable "state of occurrence" was used, organized according to regions (North, Northeast, Southeast, South, and Midwest) and country. Population projections were made considering the proportion of women aged 18 to 59 years in rural areas for each region (country, region, state, and Federal District – DF), between 2000 and 2010. To make the proportion projections for the years 2011 to 2020, the following equation was used: π_year_=π_previous year_+(π_2010_-π_2000_)/10, where π_year_ is the proportion of women aged 18 to 59 years in rural areas in relation to the region in a given year. The final projection considers the estimates from the Brazilian Institute of Geography and Statistics (IBGE)^11^ and the proportion projections (π_year_): N_year_=π_year_×I_year_, where N_year_ is the population of women aged 18 to 59 years, from the rural area of a region in a given year; π_year_ is the proportion of women aged 18 to 59 years, from the rural area, in relation to the region in a given year; and I_year_ is the official IBGE projection of the population of a region in a given year.

### Data analysis

The sociodemographic characteristics and events were descriptively analyzed according to the most reported types of violence (physical, psychological/moral, and sexual). The homogeneity of frequencies was assessed using the χ^2^ test, considering p<0.05 and their respective 95% confidence intervals (CI).

For the response variable, that is, the notification rate, the total number of notifications of all types of violence (physical, psychological/moral, sexual, torture, financial/economic, self-inflicted, human trafficking, neglect/abandonment, and legal intervention) against adult women (aged 18 to 59 years) in rural areas in each year (from 2011 to 2020) and geographic region (state, Federal District, and region) was considered as the numerator; and, as the explanatory variable, that is, the denominator, the population of adult women in rural areas, according to geographic region, in each year, according to the calculations of population projections x100 thousand: total number of notifications of violence against adult women in rural areas in each year and geographic region/total number of adult women in rural areas in each year and geographic region x100 thousand. The annual average of notification rates was calculated using the obtained quotient, divided by the total number of years of study x100 thousand. The R program version 4.2.3 was used for the analyses.

For temporal trend analyses, the notification rates of all types of violence were used based on the inflection point regression model (joinpoint regression analysis) for violence rates, according to geographic region (state, DF, and region). With this model it is possible to evaluate whether a line with multiple segments is statistically better at describing the temporal evolution of a dataset than a straight line or one with fewer segments^12^. Thus, the values of the annual percent change (APC) and the average annual percent change (AAPC) are estimated, considering 95%CI and a 5% significance level, enabling to identify the trends: stationary (p>0.05), upward (p<0.05 and positive regression coefficient), and downward (p<0.05 and negative regression coefficient)^12,13^. The analyses were performed in the Joinpoint Regression Program, version 5.1.0.0.

### Ethical aspects

The research was approved by the Research Ethics Committee of the Escola Nacional de Saúde Pública Sergio Arouca/Fiocruz, on May 9, 2022, under opinion No. 5.395.759. The database was provided by the Brazilian Ministry of Health.

## RESULTS

A total of 79,229 thousand notifications of violence against rural women, aged 18 to 59 years, in Brazil, were reported from 2011 to 2020, of which 60,819 (77.6%) were physical violence, 28,544 (36.5%) psychological/moral violence, and 4,873 (6.2%) sexual violence. In Table 1, all variables were statistically significant (p<0.05). Regarding age, in physical, psychological/moral, and sexual violence, notifications of women aged 18 to 29 and 30 to 39 years predominated. As for ethnicity/skin color, in the three types of violence, notifications of brown/Black women were the majority. Concerning marital status, married women accounted for half or more of the reports of psychological/moral and physical violence, followed by single women. As for sexual violence, single women accounted for the majority of notifications, followed by married women. Regarding level of education, women who were illiterate/had some elementary school represented the majority of notifications in all types of violence.

**Table 1:** Frequency of notifications of violence against women living in rural contexts, according to women's characteristics and types of violence. Brazil, 2011– 2020.

Characteristics		Physical violence*			Psychological violence*			Sexual violence*		
		n	(%)	(95%CI)	n	(%)	(95%CI)	n	(%)	(95%CI)
Age (years)										
	18–29	24,861	(40.9)	(40.5–41.3)	10,988	(38.5)	(37.9–39.1)	2,524	(51.8)	(50.4–53.2)
	30–39	18,865	(31.0)	(30.7–31.4)	9,076	(31.8)	(31.3–32.3)	1,233	(25.3)	(24.1–26.6)
	40–49	11,393	(18.7)	(18.4–19)	5,524	(19.3)	(18.9–19.8)	734	(15.1)	(14.1–16.1)
	50–59	5,700	(9.4)	(9.1–9.6)	2,956	(10.4)	(10–10.7)	382	(7.8)	(7.1–8.6)
Ethnicity/skin color										
	White	19,813	(33.0)	(32.6–33.4)	10,391	(36.7)	(36.1–37.2)	1,482	(30.7)	(29.4–32)
	Black/brown	31,730	(52.8)	(52.4–53.2)	14,941	(52.7)	(52.2–53.3)	2,916	(60.3)	(59–61.7)
	Asian	444	(0.7)	(0.7–0.8)	206	(0.7)	(0.6–0.8)	39	(0.8)	(0.6–1.1)
	Indigenous	4,268	(7.1)	(6.9–7.3)	1,352	(4.8)	(4.5–5)	193	(4.0)	(3.5–4.6)
	Unknown	3,832	(6.4)	(6.2–6.6)	1,435	(5.1)	(4.8–5.3)	202	(4.2)	(3.6–4.8)
Marital status										
	Single	16,383	(27.5)	(27.2–27.9)	7,183	(25.6)	(25.1–26.2)	2318	(48.5)	(47.1–49.9)
	Married	32,530	(54.7)	(54.3–55.1)	16,690	(59.6)	(59–60.2)	1752	(36.6)	(35.3–38)
	Widow	833	(1.4)	(1.3–1.5)	433	(1.5)	(1.4–1.7)	92	(1.9)	(1.6–2.4)
	Separated	3,375	(5.7)	(5.5–5.9)	2031	(7.3)	(7–7.6)	290	(6.1)	(5.4–6.8)
	Unknown	6,379	(10.7)	(10.5–11)	1667	(6.0)	(5.7–6.2)	328	(6.9)	(6.2–7.6)
Level of education										
	Illiterate/SES	22,727	(40.8)	(40.4–41.2)	11,751	(44.2)	(43.6–44.8)	1,849	(40.6)	(39.2–42.1)
	ES/SHS	9,467	(17.0)	(16.7–17.3)	4,995	(18.8)	(18.3–19.3)	829	(18.2)	(17.1–19.4)
	HS/SCE	8,532	(15.3)	(15–15.6)	4,538	(17.1)	(16.6–17.5)	850	(18.7)	(17.6–19.9)
	CE	759	(1.4)	(1.3–1.5)	539	(2.0)	(1.9–2.2)	85	(1.9)	(1.5–2.3)
	Unknown	14,221	(25.5)	(25.2–25.9)	4,759	(17.9)	(17.4–18.4)	935	(20.6)	(19.4–21.8)

Source: Notifiable Diseases Information System (SINAN).

* χ^2^ test: all variables were statistically significant (p<0.05). n: absolute number; 95%CI: 95% confidence interval; SES: some elementary school; ES: elementary school; SHS: some high school; HS: high school; SCE: some college education; CE: college education.

In Table 2, all variables were statistically significant (p<0.05). The residence was the main place where physical, psychological/moral, and sexual violence occurred. In notifications of sexual violence, in addition to the residence, the street and other locations are also significant. Recurrence was higher in physical and psychological/moral violence than in sexual violence. In the three types of violence, regarding the sex of the probable aggressor, the predominance was man and the number of people involved was one person. The largest proportion of notifications of physical and psychological/moral violence were by the spouse/boyfriend; in the case of sexual violence, the majority were by strangers, followed by friends/acquaintances, and spouse or boyfriend. The health network was the predominant referral location for the three cases of violence, followed by referrals to other police stations.

**Table 2:** Frequency of notifications of violence against women living in rural contexts, according to characteristics of the place of occurrence, likely aggressors, referral location, and types of violence suffered. Brazil, 2011– 2020.

Characteristics		Physical violence*			Psychological violence*			Sexual violence*		
		n	(%)	(95%CI)	n	(%)	(95%CI)	n	(%)	(95%CI)
Place of occurrence										
	Residence	43,811	(72.5)	(72.1–72.8)	22,303	(78.4)	(77.8–78.8)	2,778	(57.2)	(55.8–58.6)
	The street	5,084	(8.4)	(8.2–8.6)	2432	(8.5)	(8.2–8.9)	827	(17.1)	(16–18.1)
	Other places	7,079	(11.7)	(11.5–12)	2,964	(10.4)	(10.1–10.8)	963	(19.8)	(18.7–21)
	Unknown	4,472	(7.4)	(7.2–7.6)	774	(2.7)	(2.5–2.9)	287	(5.9)	(5.3–6.6)
Recurrence										
	Yes	26,577	(44.3)	(43.9–44.7)	16,439	(58.0)	(57.5–58.6)	1,706	(35.4)	(34–36.7)
	No	24,062	(40.1)	(39.7–40.5)	9,527	(33.6)	(33.1–34.2)	2,666	(55.2)	(53.8–56.7)
	Unknown	9,329	(15.6)	(15.3–15.8)	2,360	(8.4)	(8–8.7)	454	(9.4)	(8.6–10.3)
Sex of the likely aggressor										
	Man	42,018	(69.9)	(69.6–70.3)	23,232	(82.1)	(81.6–82.5)	4,670	(96.2)	(95.6–96.7)
	Woman	12,432	(20.7)	(20.4–21)	3,682	(13.0)	(12.6–13.4)	47	(1.0)	(0.7–1.3)
	Both sexes	1,135	(1.9)	(1.8–2)	621	(2.2)	(2–2.4)	24	(0.5)	(0.3–0.7)
	Unknown	4,499	(7.5)	(7.3–7.7)	768	(2.7)	(2.5–2.9)	112	(2.3)	(1.9–2.8)
Number of people involved										
	One	43,497	(73.2)	(72.8–73.5)	21,016	(75.1)	(74.6–75.6)	3,771	(77.7)	(76.5–78.9)
	Two or more	12,147	(20.4)	(20.1–20.8)	6,322	(22.6)	(22.1–23.1)	860	(17.7)	(16.7–18.8)
	Unknown	3,790	(6.4)	(6.2–6.6)	641	(2.3)	(2.1–2.5)	220	(4.6)	(4–5.2)
Relationship with the likely aggressor†										
	Spouse/boyfriend	25,557	(42.4)	(42–42.7)	14,181	(50.0)	(49.5–50.6)	905	(18.6)	(17.5–19.7)
	Former spouse/Ex-boyfriend	5,241	(8.7)	(8.5–8.9)	3,629	(12.8)	(12.4–13.2)	339	(7.0)	(6.3–7.7)
	Relative	4,953	(8.2)	(8.0–8.4)	2,584	(9.1)	(8.8–9.5)	330	(6.8)	(6.1–7.5)
	Friends/acquaintances	6,786	(11.3)	(11.0–11.5)	2,923	(10.3)	(10–10.7)	1,124	(23.1)	(21.9–24.3)
	Stranger	3,662	(6.1)	(5.9–6.3)	1,510	(5.3)	(5.1–5.6)	1,602	(32.9)	(31.6–34.3)
	Others	544	(0.9)	(0.8–1.0)	334	(1.2)	(1.1–1.3)	75	(1.5)	(1.2–1.9)
Referral location†										
	Health network	32,588	(56.0)	(55.6–56.4)	14,040	(51.1)	(50.5–51.7)	2,772	(59.7)	(58.2–61.1)
	Social welfare network	6,131	(13.8)	(13.5–14.2)	4,144	(19.9)	(19.4–20.5)	839	(24.5)	(23.1–26)
	Other police stations	15,239	(34.4)	(33.9–34.8)	7,925	(38.1)	(37.4–38.8)	1,122	(32.8)	(31.3–34.4)
	Women's Police Station	4,486	(10.1)	(9.8–10.4)	3,075	(14.8)	(14.3–15.3)	777	(22.7)	(21.4–24.2)
	Office on Violence Against Women	1,749	(3.9)	(3.8–4.1)	1,474	(7.1)	(6.8–7.5)	491	(14.4)	(13.2–15.6)
	Public Prosecutor's Office	383	(0.9)	(0.8–1.0)	307	(1.5)	(1.3–1.7)	76	(2.2)	(1.8–2.8)
	Human Rights Reference Center‡	98	(0.2)	(0.2–0.3)	61	(0.3)	(0.2–0.4)	18	(0.5)	(0.3–0.8)
	Public Defender's Office	313	(0.7)	(0.6–0.8)	332	(1.6)	(1.4–1.8)	61	(1.8)	(1.4–2.3)

Source: Notifiable Diseases Information System (SINAN)

* χ^2^ test: all variables were statistically significant (p<0.05);

† it does not add up to 100%, as this is a variable with multiple responses;

‡ data included in notification forms only as of 2015; n: absolute number; 95%CI: 95% confidence interval.

In Table 3 we can observe that in all states there was growth over the period. Considering 2020, the first year of the new coronavirus (COVID-19) pandemic and social restrictions, we observed a slight decrease in the rates in 17 states, such as Amapá, Tocantins, most states in the Northeast, all in the South region, and Goiás. This decrease was reflected in the country's rate, which dropped from 162.8 in 2019 to 142.6 in 2020.

**Table 3:** Annual rate of notifications of violence against women living in rural contexts in the Notifiable Diseases Information System, according to country, region, states, and the Federal District. Brazil, 2011– 2020.

Country, region, states, and DF		2011	2012	2013	2014	2015	2016	2017	2018	2019	2020	Average rate
Brazil		36.7	56.2	76.1	90.4	105.2	101.0	123.8	143.3	162.8	142.6	103.8
North		12.1	23.1	35.6	42.6	54.3	54.7	65.1	78.0	89.4	93.7	54.9
	Rondônia	9.4	15.2	15.4	41.8	33.5	44.0	61.0	86.7	71.4	90.9	46.9
	Acre	31.3	75.7	64.4	73.0	99.3	125.6	169.5	176.0	127.5	178.1	112.0
	Amazonas	14.8	42.0	82.7	75.0	121.5	128.0	131.4	157.2	200.9	209.3	116.3
	Roraima	49.1	104.5	127.4	149.1	117.1	172.9	263.9	263.8	277.4	297.9	182.3
	Pará	5.3	8.8	17.2	19.7	28.4	19.5	23.8	30.4	41.8	43.2	23.8
	Amapá	13.6	6.6	51.2	149.5	115.1	82.6	80.5	123.4	186.1	122.9	93.1
	Tocantins	41.2	55.2	62.1	85.9	91.3	117.7	144.2	170.5	167.4	153.3	108.9
Northeast		23.7	30.4	44.8	49.8	56.9	51.7	66.9	83.6	100.3	93.0	60.1
	Maranhão	5.0	12.8	27.5	25.7	22.8	16.5	23.4	30.4	38.6	37.4	24.0
	Piauí	21.5	30.6	49.1	41.4	52.6	68.5	83.9	75.8	89.1	63.3	57.6
	Ceará	12.1	22.6	26.3	28.4	39.7	41.6	61.3	87.6	113.7	111.0	54.4
	Rio Grande do Norte	18.4	28.6	38.4	45.9	44.3	41.1	58.5	81.4	111.4	87.6	55.6
	Paraíba	9.4	15.9	17.2	20.0	37.2	28.6	38.0	48.6	63.3	65.7	34.4
	Pernambuco	64.6	74.0	104.0	116.9	117.5	101.3	144.9	171.7	203.7	173.3	127.2
	Alagoas	95.7	91.6	121.0	124.1	156.7	123.8	154.6	219.2	204.9	192.5	148.4
	Sergipe	5.5	9.5	30.1	47.5	57.8	44.9	36.1	56.1	91.1	82.6	46.1
	Bahia	15.2	19.6	31.6	41.1	47.9	45.9	52.7	63.5	76.1	80.9	47.4
Southeast		67.6	117.8	163.2	214.9	261.5	256.1	297.2	336.4	357.3	284.2	235.6
	Minas Gerais	66.3	118.1	213.4	286.5	351.6	321.6	332.0	359.8	368.0	254.4	267.2
	Espírito Santo	20.2	37.4	71.1	94.5	143.2	168.7	201.9	277.3	317.4	301.1	163.3
	Rio de Janeiro	80.7	121.0	125.6	139.0	124.1	128.2	193.1	212.1	241.7	239.8	160.5
	São Paulo	83.3	148.6	121.0	155.1	184.5	211.0	318.9	381.6	428.8	409.7	244.3
South		60.4	94.8	103.5	115.2	128.1	134.0	180.0	204.2	250.7	213.9	148.5
	Paraná	35.6	64.3	99.7	106.1	117.6	138.8	183.1	189.5	213.1	211.3	135.9
	Santa Catarina	67.2	86.1	86.4	91.3	96.6	98.4	141.5	152.1	187.4	143.9	115.1
	Rio Grande do Sul	80.2	130.4	118.6	140.0	159.3	153.1	202.9	254.2	331.7	264.4	183.5
Midwest		33.1	42.6	80.1	86.3	92.0	80.4	106.5	119.1	141.4	155.1	93.7
	Mato Grosso do Sul	99.7	106.5	226.3	249.1	229.0	177.8	267.4	305.9	367.6	382.5	241.2
	Mato Grosso	11.1	15.9	24.7	44.8	49.7	42.0	50.2	51.1	48.1	74.2	41.2
	Goiás	10.7	20.2	35.5	28.0	48.4	48.7	56.6	58.3	79.5	77.5	46.3
	Federal District	60.7	109.9	151.7	90.7	101.4	150.2	148.4	205.3	235.0	261.3	151.5

Source: Notifiable Diseases Information System and population projections based on the demographic census of 2000 and 2010, from the Brazilian Institute of Geography and Statistics, of women living in rural regions aged 18 to 59 years. DF: Federal District.

In the APC, the notification rates of violence against rural women in Brazil, according to the regression model, had two inflection points, and the trends were classified as upward. The North and Midwest regions had two inflection points (2011–2013 and 2013–2020), classified as upward; the Southeast had an increasing inflection point in the first four years and then stationary; the South and Northeast had an inflection point demonstrating continuous increase throughout the period. Regarding the states (18) and the DF, there were one and two inflection points with an upward trend, five with an upward trend in the first three/four years, and then stationary in the remaining period. Pará was the only state with a stationary trend from 2011 to 2013, and an upward trend for the remaining period; and Goiás was the only one that only had a stationary trend at three inflection points. In the AAPC, all country, region, states, and DF rates showed statistically significant increasing temporal behavior (Table 4).

**Table 4:** Temporal trend with the annual percent change and the average annual percent change, by joinpoint regression, of the notification rates of violence against women living in rural contexts in the Notifiable Diseases Information System, according to country, region, states, and the Federal District. Brazil, 2011– 2020.

Country, region, states, and DF		Period	APC (95%CI)	Classification	AAPC (95%CI)	Classification
Brazil		2011–2013	**47.7** * (6.1–105.7)	Upward	**17.9** * (10.8–25.4)	Upward
		2013–2020	**10.5** * (5.7–15.5)	Upward		
North		2011–2013	**74.2** * (43.2– 112.1)	Upward	**25.9** * (21.3–30.6)	Upward
		2013–2020	**14.7** * (11.7–17.8)	Upward		
	Rondônia	2011–2020	**28.4** * (20.2–37.2)	Upward	**28.4** * (20.2–37.2)	Upward
	Acre	2011–2020	**17.8** * (10.3–25.9)	Upward	**17.8** * (10.3–25.9)	Upward
	Amazonas	2011–2013	**125.0** * (46.5–245.6)	Upward	**33.4** * (23.1–44.6)	Upward
		2013–2020	**14.9** * (8.5–21.7)	Upward		
	Roraima	2011–2020	**19.1** * (12.4–26.1)	Upward	**19.1** * (12.4–26.1)	Upward
	Pará	2011–2013	80.8 (-9.3–260.6)	Stationary	**26.1** * (10.8–43.5)	Upward
		2013–2020	**13.7** * (3.7–24.7)	Upward		
	Amapá	2011–2020	**31.7** * (8.8–59.4)	Upward	**31.7** * (8.8–59.4)	Upward
	Tocantins	2011–2018	**22.1** * (18.8–25.5)	Upward	**15.2** * (10.9–19.7)	Upward
		2018–2020	-5.9 (-23.3–15.5)	Stationary		
Northeast		2011–2020	**16.1** * (12.2–20.0)	Upward	**16.1** * (12.2–20.0)	Upward
	Maranhão	2011–2013	**138.4** * (21.5–368.0)	Upward	**26.5** * (11.0–44.1)	Upward
		2013–2016	-13.8 (-56.1–69.3)	Stationary		
		2016–2020	22.8 (-0.8–52.0)	Stationary		
	Piauí	2011–2020	**14.1** * (7.4–21.2)	Upward	**14.1** * (7.4–21.2)	Upward
	Ceará	2011–2013	37.0 (-11.7–112.7)	Upward	**27.9** * (17.8–38.9)	Upward
		2013–2020	**25.5** * (18.3–33.1)	Upward		
	Rio Grande do Norte	2011–2020	**18.5** * (12.9–24.3)	Upward	**18.5** * (12.9–24.3)	Upward
	Paraíba	2011–2020	**22.9** * (17.9–28.1)	Upward	**22.9** * (17.9–28.1)	Upward
	Pernambuco	2011–2020	**12.2** * (8.3–16.2)	Upward	**12.2** * (8.3–16.2)	Upward
	Alagoas	2011–2020	**9.7** * (6.2–13.4)	Upward	**9.7** * (6.2–13.4)	Upward
	Sergipe	2011–2014	**101.7** * (19.7–239.7)	Upward	**33.6** * (13.9–56.8)	Upward
		2014–2020	8.7 (-8.8–29.7)	Stationary		
	Bahia	2011–2014	**39.3** * (21.1–60.1)	Upward	**20.4** * (15.4–25.7)	Upward
		2014–2020	**12.0** * (6.9–17.4)	Upward		
Southeast		2011–2014	**49.2** * (21.3–83.5)	Upward	**18.7** * (11.4–26.5)	Upward
		2014–2020	5.9 (-1.2–13.6)	Stationary		
	Minas Gerais	2011–2014	**69.9** * (36.3–111.7)	Upward	**18.4** * (10.7–26.7)	Upward
		2014–2020	-1.1 (-8.2–6.5)	Stationary		
	Espírito Santo	2011–2013	**92.2** * (8.8–239.6)	Upward	**35.7** * (23.6–49.0)	Upward
		2013–2018	**31.1** * (9.5–56.9)	Upward		
		2018–2020	4.4 (-40.9–84.5)	Stationary		
	Rio de Janeiro	2011–2020	**11.7** * (8.0–15.6)	Upward	**11.7** * (8.0–15.6)	Upward
	São Paulo	2011–2020	**19.8** * (15.3–24.5)	Upward	**19.8** * (15.3–24.5)	Upward
South		2011–2020	**14.9** * (11.4–18.5)	Upward	**14.9** * (11.4–18.5)	Upward
	Paraná	2011–2013	**64.1** * (20.7–123.0)	Upward	**22.7** * (15.9–30.0)	Upward
		2013–2020	**12.9** * (8.4–17.7)	Upward		
	Santa Catarina	2011–2020	**10.5** * (7.0–14.1)	Upward	**10.5** * (7.0–14.1)	Upward
	Rio Grande do Sul	2011–2020	**14.4** * (10.3–18.6)	Upward	**14.4** * (10.3–18.6)	Upward
Midwest		2011–2013	**52.4** * (5.0–121.2)	Upward	**18.9** * (10.9–27.4)	Upward
		2013–2020	**10.7** * (5.3–16.4)	Upward		
	Mato Grosso do Sul	2011–2020	**14.4** * (7.9–21.4)	Upward	**14.4** * (7.9–21.4)	Upward
	Mato Grosso	2011–2014	**57.7** * (22.2–103.5)	Upward	**21.5** * (12.4–31.4)	Upward
		2014–2020	6.7 (-2.1–16.3)	Stationary		
	Goiás	2011–2013	70.1 (-44.4–420.6)	Stationary	**24.7** * (4.1–49.5)	Upward
		2013–2017	14.8 (-34.4–100.9)	Stationary		
		2017–2020	13.3 (-35.2–98.2)	Stationary		
	Federal District	2011–2020	**14.2** * (7.7–21.0)	Upward	**14.2** * (7.7–21.0)	Upward

Source: Notifiable Diseases Information System. DF: Federal District; APC: annual percent change; 95%CI: 95% confidence interval; AAPC: average annual percent change.

* p<0.05. Bold values indicate statistically significant results.

## DISCUSSION

Among the characteristics of notifications of physical, psychological/moral, and sexual violence against rural women, we observed a higher frequency among women aged 18 to 39 years, brown/Black, married, single, and with low levels of education. The residence was identified as the main place where violence occurred, which was recurrent, especially in physical, psychological/moral terms, and was mostly perpetrated by a single man. The aggressor, in most cases, was the spouse/boyfriend, but in sexual cases known and unknown aggressors were also identified. Mostly, notification rates of violence against rural women increased throughout the studied period and, in 2020, the first year of the COVID-19 pandemic, there was no significant decrease.

The characteristics of women highlighted in this study corroborate other research^1,14^. Intentional violent deaths (IVD) especially victimize young women, and femicide occurred in practically all age groups, but with a higher prevalence of deaths throughout women's reproductive lives. Among the biggest victims of femicide and IVD are Black women. Furthermore, in femicides, the main perpetrator is the victim's partner or ex-partner, and the residence is the main place of occurrence^12^. Femicide is the ultimate expression of gender-based violence, and researchers emphasize that women with prior notification of violence are at greater risk of femicide compared to the general female population^14,15^.

Regarding low levels of education, in the case of rural women, according to the Agricultural Census (2017), more than 60.0% of them had completed elementary school. Rural women often drop out of school due to the need to help with family work, poor conditions/lack of school transport, and early marriage. The latter is more pronounced in rural areas in Brazil^15^. Early marriage is often motivated by the young woman's search for more freedom to leave home and have a better life, without violence or poverty. But what happens is that the teenager becomes pregnant and drops out of school, reducing her chances of formal employment, becoming dependent on her partner, limited to domestic services, and vulnerable to violence^16^. This datum is reinforced by IBGE, which identified, in 2023, the need for work, domestic chores/caring for people, and pregnancy as the main reasons for school dropout among women aged 15 to 29 years, especially among Black and brown women^17^.

Physical violence was also the most mentioned in other studies on rural women^18,19^, which may be due to the fact that this violence is more known and recognized and generates visible injuries that require treatment in health services^20^. Authors of a study conducted with rural workers in the state of Pernambuco, Brazil, on their perception of violence, showed that a large proportion of these women considered violence only to be events that left physical marks^19^. Conversely, psychological violence may be more present in women's lives, but it may be underreported because it is a type of violence that is difficult for women themselves or health professionals to identify^20,21^.

As for sexual violence, the results draw attention to young, single, Black/brown women, whose aggressors are strangers, acquaintances, or male partners, and it often happens once. There is still a naturalization in society of sexual violence in certain situations, whether because the partner believes he has "conjugal rights" over the woman, or because strange men, or even acquaintances, are supported by a patriarchal and sexist culture of control and imposition of power over female bodies, evidencing the lack of safety for women inside and outside their homes^21^. Furthermore, Black women's lives have been marked by several types of violence over the centuries. The intersectional perspective contributes to the discussion of violence that permeates the dimensions of gender, race, and class, which increase the vulnerabilities and inequalities experienced by Black women^22,23^.

The results may still be underestimated, as women have difficulty seeking assistance services due to fear of judgment, feelings of guilt, or shame. When sexual violence occurs between intimate partners, women also tend to remain silent due to cultural, religious, and social beliefs, which keep intimate relationship issues in the private domain^24^.

Violence against rural women was shown to be mostly recurrent and to occur in their own residences. Often, violence does not begin with a serious assault, but it is masked by subtle forms of violence, such as "overprotection" and "jealousy," which make the woman feel guilty and begin to tolerate the assaults — which, in turn, paves the way for other types of violence (physical, patrimonial, sexual) and their repetition^25,26^. In this case, the home becomes a place of affection and tension in which the woman isolates herself out of fear or shame, feels lonely and, over time, can develop low self-esteem, anxiety, emotional dependence, depression, etc.^27^.

The health network was the main referral location. This may occur because it is usually sought after in the most serious situations of physical or sexual violence, or when mental health is already severely affected, in such a way that other referrals for treatment or monitoring are necessary^4,28^. The other locations were police stations and the social welfare network, which generally exist even in small municipalities. Conversely, women's police stations and the Office on Violence Against Women tend to be concentrated in larger cities. According to *Revista AzMina* [AzMina Magazine]^29^, until 2020, only 7.0% of Brazilian cities had a Women's Police Station. In small municipalities, only nine of the 3,600 cities with up to 20 thousand inhabitants had a Women's Police Station. In other words, women end up looking for regular police stations, where they are not always welcome, and suffer another type of violence: institutional violence^22^.

Regarding the increase in the annual rates of violence notification in the states, one explanation for this scenario may be the improvement in epidemiological surveillance over the years. Since the universalization of notification of interpersonal/self-inflicted violence in 2011, there has been a substantial increase in SINAN coverage in Brazil. For instance, the country ranged from 2,114 notifying municipalities (38.0%) in 2011 to 4,381 notifying municipalities (78.7%) in 2018. The higher rates in the Southeast are related to the better functioning of surveillance (89.4% coverage in 2018)^30^.

It is also worth noting the decrease in notification rates during the first year of the COVID-19 pandemic, possibly influenced by the imposition of social isolation. Conversely, this decrease was not statistically significant, which perhaps indicates the severity of the cases of violence that led these women, in the midst of a pandemic, to leave their homes and reach out the health sector.

Despite the increase in notification coverage, underreporting still persists. Difficulties in recognizing cases of violence and lack of training among professionals to recognize and report violence are some of the existing limitations^15,31–33^. Furthermore, the health sector is still not fully recognized as a gateway to the Office on Violence Against Women. When women seek support, they generally turn first to people they trust, and when violence becomes extreme, such as in cases of physical injuries or threats, they seek out the health sector or police stations^4,7,28,34^.

Another aspect is small municipalities, where women may feel embarrassed to seek health services to report the violence they have suffered, remaining in violent relationships for a long time. Professionals, in turn, may have difficulty reporting for fear of reprisals from the aggressor^19,32^.

In order to take effective action to combat violence, it is necessary to consider the specificities of the rural context: lack of information, absence of telephone and Internet services, geographic isolation, lack of public transport, and long distances to urban areas and between neighbors/family members^4–6,35^. Interventions are needed in the health sector, such as more training for health professionals in the process of identifying, reporting, and supporting rural women in situations of violence, especially community health agents, who are closest to women; integration of health, assistance, and security services, as the issue of violence requires intersectional and comprehensive care, which is also present in rural areas.

The limitations of this study are inherent in the use of secondary data, as some records may present low completeness and underreporting. Vasconcelos et al.^34^ estimated that, in 2019, underreporting of violence against women in Brazil was 98.5, 75.9, and 89.4% for psychological, physical, and sexual violence, respectively. The same woman may appear more than once on the database, but as the database is not identified, repetitions could not be excluded.

The results presented in this article help to identify the main characteristics of women and violent events. Reported cases of violence against rural women increased between 2011 and 2020 throughout the country. Furthermore, the greater vulnerability of young, Black, married or single women with low levels of education stands out. We emphasize that actions to combat the violence must consider that violence occurs mainly by people close to women's relationships and in the home itself, which makes it more difficult for individuals themselves to speak out and for health professionals to identify the violence. Strengthening the training of health professionals is encouraged to improve and expand the notification process as a tool for caring for women in situations of violence, considering rural contexts and their specificities.
