# Supplementary material for: Characterization of notifications of violence against women living in rural contexts in Brazil from 2011 to 2020
Source: Rev Bras Epidemiol. 2024 Dec 13;27:e240059. doi: 10.1590/1980-549720240059 (PMC11654865; doi:10.1590/1980-549720240059)
Supplement: Translation

# Translation: Caracterização das notificações de violência contra as mulheres que vivem em contextos rurais no Brasil, de 2011 a 2020

**DOI:** 10.1590/1980-549720240059.2

**Authors:** Luciane Stochero, Liana Wernersbach Pinto

**Affiliations:** I Fundação Oswaldo Cruz, Escola Nacional de Saúde Pública Sergio Arouca – Rio de Janeiro (RJ), Brasil.

**Keywords:** Violência contra a mulher, Violência de gênero, População rural, Sistemas de informação em saúde, Notificação

## Abstract

**Objetivo:**

Descrever e analisar as notificações e a tendência temporal da violência contra as mulheres que vivem em contextos rurais no Brasil, no período de 2011 a 2020.

**Métodos:**

Estudo ecológico de série temporal de caráter descritivo e analítico, com dados do Sistema de Informação de Agravos de Notificação (SINAN) sobre violência contra as mulheres com idade de 18 a 59 anos, da zona rural, de 2011 a 2020, no Brasil. As análises foram descritivas e de tendência, com o modelo de regressão por pontos de inflexão (*joinpoint*) e cálculo da variação percentual anual (VPA) e variação média no período (VMP).

**Resultados:**

Foram registradas 79.229 notificações de violência contra mulheres rurais. As violências mais notificadas foram a física (77,6%), a psicológica/moral (36,5%) e a sexual (6,2%), sobretudo, entre mulheres jovens, negras, casadas e de baixa escolaridade. Foram cometidas, na maioria dos casos, na residência e, principalmente, pelo companheiro do sexo masculino. A VPA foi estatisticamente crescente ao longo do período estudado no Brasil, nas regiões Norte, Centro-Oeste, Sul e Nordeste, envolvendo18 estados e o Distrito Federal (DF). Na VMP, todas as taxas de país, regiões, estados e DF apresentaram tendência estatisticamente crescente.

**Conclusão:**

Diante dos crescentes casos notificados de violência contra as mulheres rurais em todo o país, que ocorrem, principalmente, por pessoas de sua relação e no próprio lar, incentiva-se o reforço na capacitação dos profissionais de saúde para melhoria e ampliação do processo de notificação como instrumento de cuidado com as mulheres em situação de violência.

## INTRODUÇÃO

A violência contra as mulheres é compreendida como qualquer ação ou conduta, baseada no gênero, inclusive decorrente de discriminação ou desigualdade étnica, que cause sua morte, dano ou sofrimento físico, sexual ou psicológico^1^. No Brasil, estima-se que, em média, 18,6 milhões de mulheres, a partir de 16 anos, sofreram alguma forma de violência ao longo de 2022; as mais frequentes foram: ofensas verbais, perseguição, ameaças e agressão física^2^.

Os dados, como a maioria das pesquisas, não diferenciam mulheres residentes no meio urbano e rural, e essa ausência de informações relativas às mulheres que vivem em contextos rurais contribuiu para a invisibilidade da problemática e, consequentemente, menos ações de enfrentamento da violência nesse contexto^3^.

O contexto rural abarca uma série de particularidades, como ausência de vizinhos e familiares, falta de autonomia financeira, isolamento social e geográfico, afastamento dos serviços de saúde e proteção, que normalmente se localizam nos centros urbanos^4–6^. Com isso, "as mulheres rurais encontram potencialização em adversidades como a exclusão e as dificuldades de acesso a serviços de saúde e segurança"^4^. Essas adversidades, enraizadas na opressão de gênero, colaboram para fortalecer o silenciamento dessas mulheres.

Entre as poucas publicações sobre a violência contra as mulheres que vivem em contextos rurais, destaca-se uma pesquisa com dados do Sistema de Informação de Agravos de Notificação (SINAN), de 2010 a 2012, com 7.197 notificações contra mulheres rurais brasileiras adultas. Os tipos de violência mais notificados foram a física (76,8%), a psicológica/moral (38,4%) e a sexual (7,4%)^2^. Outro estudo mais recente, com dados da Pesquisa Nacional de Saúde (2019), observou a vivência de violência psicológica (18,0%), física (4,4%) e a sexual (1,5%), nos últimos 12 meses, por mulheres da zona rural, de 18 a 59 anos de idade^7^. Em ambas as pesquisas, os agressores, em sua maioria, eram pessoas conhecidas, e a residência foi o principal local de ocorrência^7^.

O setor da saúde tem importante papel no enfrentamento da violência contra as mulheres^4,8^. Os profissionais de saúde são responsáveis por identificar, acolher e escutar atenta e cuidadosamente, orientar, acompanhar e encaminhar os casos de violência, bem como registrá-los em prontuário e notificá-los^8^. A notificação constitui um elemento-chave para a atenção integral às mulheres, retirando os casos de violência da invisibilidade, prevenindo a violência de repetição e permitindo que a rede de proteção e de garantia de direitos seja acionada e se articule^9^.

A violência contra as mulheres rurais é um problema de saúde pública. Os contextos rurais podem potencializar a violência, impedir o pedido de ajuda, fazendo com que as mulheres permaneçam mais tempo em situação de violência. Os estudos de abrangência nacional nessa temática ainda são escassos, invisibilizando o problema. O presente estudo pretende colaborar para preencher essa lacuna e ampliar o conhecimento dessa temática. Seu objetivo é descrever e analisar as notificações e a tendência temporal da violência contra as mulheres que vivem em contextos rurais no Brasil, no período de 2011 a 2020.

## MÉTODOS

### Delineamento do estudo

Trata-se de um estudo ecológico de série temporal de caráter descritivo e analítico, utilizando como fonte de dados as fichas de notificação do SINAN sobre violência contra mulheres com idade de 18 a 59 anos, pertencentes à zona rural, no período de 2011 a 2020, de todas as unidades federativas (UFs) do Brasil. A escolha do início do período de análise foi em razão da Portaria n^o^ 104/2011, que definiu a violência doméstica e sexual como agravo de notificação compulsória e universalizou as notificações de violência para todos os serviços de saúde^9^.

### Variáveis do estudo

Tipos de violência: física, psicológica/moral, sexual, tortura, financeira/econômica, autoprovocada, tráfico de seres humanos, negligência/abandono, violência por intervenção legal, outras^10^.

No campo "zona de residência", foi utilizada a opção rural, definida como "área com características próprias do campo, com população dispersa, relativamente distante dos centros administrativos, acesso limitado a serviços públicos, agroprodução"^10^.

Variáveis sociodemográficas: idade (18–29, 30–39, 40–49 e 50–59), raça/cor (branca, parda/preta, indígena, amarela e ignorada), estado civil (solteira, casada/união consensual, viúva, separada, ignorado) e escolaridade (sem instrução/ensino fundamental incompleto, ensino fundamental completo/ensino médio incompleto, ensino médio completo/ensino superior incompleto e ensino superior completo).

Variáveis das características dos eventos: local de ocorrência (residência/habitação coletiva, via pública, outros locais (escola, local de prática esportiva, bar/similar, comércio/serviços, indústrias/construção, outro), ignorado) e recorrência (sim, não, ignorado).

Variáveis das características do agressor: sexo do provável autor da agressão (masculino, feminino, ambos os sexos, ignorado), número de envolvidos (um, dois ou mais, ignorado) e vínculo com o provável autor da agressão (cônjuge/namorado, ex-cônjuge/ex-namorado, desconhecido, familiar (pai, mãe, madrasta, padrasto, filho, irmão), amigos/conhecidos, outros (pessoa com relação institucional, patrão/chefe, policial/agente da lei)).

Variáveis do local de encaminhamento: rede da saúde, rede da assistência social, rede de atendimento à mulher, ministério público, delegacia de atendimento à mulher, outras delegacias, centro de referência dos direitos humanos e defensoria pública.

Para a análise temporal, foi utilizada a variável "UF de ocorrência", organizada de acordo com as regiões (Norte, Nordeste, Sudeste, Sul e Centro-Oeste) e país. As projeções populacionais foram feitas considerando a proporção de mulheres de 18 a 59 anos da zona rural para cada região (país, região, UF e Distrito Federal (DF)), entre os anos de 2000 e 2010. Para fazer as projeções de proporção para os anos de 2011 a 2020, foi utilizada a equação: π_ano_=π_ano anterior_+(π_2010_-π_2000_)/10, em que π_ano_ é a proporção de mulheres de 18 a 59 anos da zona rural com relação à região em determinado ano. A projeção final considera as estimativas do Instituto Brasileiro de Geografia e Estatística (IBGE)^11^ e as projeções de proporção (π_ano_): N_ano_=π_ano_×I_ano_, em que N_ano_ é a população de mulheres de 18 a 59 anos, da zona rural de uma região em determinado ano; π_ano_ é a proporção de mulheres de 18 a 59 anos, da zona rural, com relação à região em determinado ano, e I_ano_ é a projeção oficial do IBGE da população de uma região em determinado ano.

### Análise dos dados

Analisou-se descritivamente as características sociodemográficas e os eventos, segundo os tipos de violência mais notificados (física, psicológica/moral e sexual). A homogeneidade das frequências foi avaliada com o teste χ^2^ (qui-quadrado), considerando o p<0,05 e seus respectivos intervalos de confiança (IC) de 95%.

Para a variável resposta, ou seja, a taxa de notificação, considerou-se como numerador o total de notificações de todos os tipos de violência (física, psicológica/moral, sexual, tortura, financeira/econômica, autoprovocada, tráfico de seres humanos, negligência/abandono e intervenção legal) contra mulheres adultas (de 18 a 59 anos de idade) da zona rural de cada ano (de 2011 a 2020) e região geográfica (UF, DF e região), e como variável explicativa, ou seja, o denominador, a população de mulheres adultas da zona rural, de acordo com a região geográfica, de cada ano, de acordo com os cálculos das projeções populacionais x100 mil: total de notificações de violência contra mulheres adultas da zona rural de cada ano e região geográfica/total de mulheres adultas da zona rural de cada ano e região geográfica x100 mil. A média anual das taxas de notificações foi calculada por meio do quociente obtido, dividido pelo total de anos do estudo x100 mil. Utilizou-se o Programa R versão 4.2.3 para as análises.

Para as análises de tendência temporal, foram utilizadas as taxas de notificação de todas as violências com base no modelo de regressão por pontos de inflexão *(joinpoint regression analysis*) para as taxas de violência, de acordo com a região geográfica (UF, DF e região). Esse modelo examina se uma linha com múltiplos segmentos é estatisticamente melhor para descrever a evolução temporal de um conjunto de dados em detrimento de uma linha reta ou com menos segmentos^12^. Assim, são estimados os valores da variação percentual anual (VPA) e a variação média do período (VMP), considerando-se IC95% e nível de significância de 5%, sendo possível identificar as tendências: estacionária (p>0,05), crescente (p<0,05 e coeficiente da regressão positivo) e decrescente (p<0,05 e coeficiente da regressão negativo)^12,13^. As análises foram realizadas no *Joinpoint Regression Program*, versão 5.1.0.0.

### Aspectos éticos

A pesquisa foi aprovada pelo Comitê de Ética em Pesquisa da Escola Nacional de Saúde Pública Sergio Arouca/Fiocruz, em 9 de maio de 2022, sob parecer n^o^ 5.395.759. O banco de dados foi cedido pelo Ministério da Saúde.

## RESULTADOS

Foram registradas 79.229 mil notificações de violência contra as mulheres rurais, com idade de 18 a 59 anos, no Brasil, de 2011 a 2020, sendo 60.819 (77,6%) de violência física, 28.544 (36,5%) psicológica/moral e 4.873 (6,2%) sexual. Na Tabela 1, todas as variáveis foram estatisticamente significativas (p<0,05). Em relação à idade, na violência física, psicológica/moral e sexual, predominaram as notificações de mulheres com 18 a 29 anos e de 30 a 39 anos. Quanto à raça/cor, nos três tipos de violência, as notificações de mulheres pardas/pretas foram maioria. No que se refere ao estado civil, as mulheres casadas representaram a metade ou mais das notificações de violência psicológica/moral e física, seguidas das solteiras. Sobre a violência sexual, as solteiras representaram a maioria das notificações, depois as casadas. Quanto à escolaridade, as mulheres que não tinham instrução/ensino fundamental incompleto representaram a maioria das notificações em todos os tipos de violência.

**Tabela 1:** Frequência das notificações de violência contra mulheres que vivem em contextos rurais, segundo as características das mulheres e os tipos de violência. Brasil, 2011–2020.

Características		Violência física*			Violência psicológica*			Violência sexual*		
		n	(%)	(IC95%)	n	(%)	(IC95%)	n	(%)	(IC95%)
Idade (anos)										
	18–29	24.861	(40,9)	(40,5–41,3)	10.988	(38,5)	(37,9–39,1)	2.524	(51,8)	(50,4–53,2)
	30–39	18.865	(31,0)	(30,7–31,4)	9.076	(31,8)	(31,3–32,3)	1.233	(25,3)	(24,1–26,6)
	40–49	11.393	(18,7)	(18,4–19)	5.524	(19,3)	(18,9–19,8)	734	(15,1)	(14,1–16,1)
	50–59	5.700	(9,4)	(9,1–9,6)	2.956	(10,4)	(10–10,7)	382	(7,8)	(7,1–8,6)
Raça/cor										
	Branca	19.813	(33,0)	(32,6–33,4)	10.391	(36,7)	(36,1–37,2)	1.482	(30,7)	(29,4–32)
	Preta/parda	31.730	(52,8)	(52,4–53,2)	14.941	(52,7)	(52,2–53,3)	2.916	(60,3)	(59–61,7)
	Amarela	444	(0,7)	(0,7–0,8)	206	(0,7)	(0,6–0,8)	39	(0,8)	(0,6–1,1)
	Indígena	4.268	(7,1)	(6,9–7,3)	1.352	(4,8)	(4,5–5)	193	(4,0)	(3,5–4,6)
	Ignorada	3.832	(6,4)	(6,2–6,6)	1.435	(5,1)	(4,8–5,3)	202	(4,2)	(3,6–4,8)
Estado civil										
	Solteira	16.383	(27,5)	(27,2–27,9)	7.183	(25,6)	(25,1–26,2)	2318	(48,5)	(47,1–49,9)
	Casada	32.530	(54,7)	(54,3–55,1)	16.690	(59,6)	(59–60,2)	1752	(36,6)	(35,3–38)
	Viúva	833	(1,4)	(1,3–1,5)	433	(1,5)	(1,4–1,7)	92	(1,9)	(1,6–2,4)
	Separada	3.375	(5,7)	(5,5–5,9)	2031	(7,3)	(7–7,6)	290	(6,1)	(5,4–6,8)
	Ignorado	6.379	(10,7)	(10,5–11)	1667	(6,0)	(5,7–6,2)	328	(6,9)	(6,2–7,6)
Escolaridade										
	Sem instrução/EFI	22.727	(40,8)	(40,4–41,2)	11.751	(44,2)	(43,6–44,8)	1.849	(40,6)	(39,2–42,1)
	EFC/EMI	9.467	(17,0)	(16,7–17,3)	4.995	(18,8)	(18,3–19,3)	829	(18,2)	(17,1–19,4)
	EMC/ESI	8.532	(15,3)	(15–15,6)	4.538	(17,1)	(16,6–17,5)	850	(18,7)	(17,6–19,9)
	ESC	759	(1,4)	(1,3–1,5)	539	(2,0)	(1,9–2,2)	85	(1,9)	(1,5–2,3)
	Ignorada	14.221	(25,5)	(25,2–25,9)	4.759	(17,9)	(17,4–18,4)	935	(20,6)	(19,4–21,8)

Fonte: Sistema de Informação de Agravos de Notificação (SINAN).

* Teste χ^2^: todas as variáveis foram estatisticamente significativas (p<0,05). n: número absoluto; IC95%: intervalo de confiança de 95%; EFI: ensino fundamental incompleto; EFC: ensino fundamental completo; EMI: ensino médio incompleto; EMC: ensino médio completo; ESI: ensino superior incompleto; ESC: ensino superior completo.

Na Tabela 2, todas as variáveis foram estatisticamente significativas (p<0,05). A residência foi o principal local de ocorrência nas violências física, psicológica/moral e sexual. Nas notificações de violência sexual, além da residência, a via pública ou outros locais também aparecem de forma expressiva. A recorrência foi maior na violência física e psicológica/moral do que na sexual. Nas três violências, quanto ao sexo do provável autor, a predominância foi o masculino e o número de envolvidos foi de uma pessoa. A maior parcela das notificações de violências física e psicológica/moral foram por parte do cônjuge/namorado, na sexual, a maioria foi por parte de desconhecido, seguido de amigos/conhecidos e cônjuge ou namorado. A rede da saúde foi o local predominante de encaminhamento nas três violências. Em seguida aparecem os encaminhamentos para outras delegacias.

**Tabela 2:** Frequência das notificações de violência contra mulheres que vivem em contextos rurais, segundo características do local de ocorrência, possíveis agressores, local de encaminhamento e tipos de violência sofrida. Brasil, 2011–2020.

Características		Violência física*			Violência psicológica*			Violência sexual*		
		n	(%)	(IC95%)	n	(%)	(IC95%)	n	(%)	(IC95%)
Local de ocorrência										
	Residência	43.811	(72,5)	(72,1–72,8)	22.303	(78,4)	(77,8–78,8)	2.778	(57,2)	(55,8–58,6)
	Via pública	5.084	(8,4)	(8,2–8,6)	2432	(8,5)	(8,2–8,9)	827	(17,1)	(16–18,1)
	Outros locais	7.079	(11,7)	(11,5–12)	2.964	(10,4)	(10,1–10,8)	963	(19,8)	(18,7–21)
	Ignorado	4.472	(7,4)	(7,2–7,6)	774	(2,7)	(2,5–2,9)	287	(5,9)	(5,3–6,6)
Recorrência										
	Sim	26.577	(44,3)	(43,9–44,7)	16.439	(58,0)	(57,5–58,6)	1.706	(35,4)	(34–36,7)
	Não	24.062	(40,1)	(39,7–40,5)	9.527	(33,6)	(33,1–34,2)	2.666	(55,2)	(53,8–56,7)
	Ignorada	9.329	(15,6)	(15,3–15,8)	2.360	(8,4)	(8–8,7)	454	(9,4)	(8,6–10,3)
Sexo do provável autor da violência										
	Masculino	42.018	(69,9)	(69,6–70,3)	23.232	(82,1)	(81,6–82,5)	4.670	(96,2)	(95,6–96,7)
	Feminino	12.432	(20,7)	(20,4–21)	3.682	(13,0)	(12,6–13,4)	47	(1,0)	(0,7–1,3)
	Ambos os sexos	1.135	(1,9)	(1,8–2)	621	(2,2)	(2–2,4)	24	(0,5)	(0,3–0,7)
	Ignorado	4.499	(7,5)	(7,3–7,7)	768	(2,7)	(2,5–2,9)	112	(2,3)	(1,9–2,8)
Número de envolvidos										
	Um	43.497	(73,2)	(72,8–73,5)	21.016	(75,1)	(74,6–75,6)	3.771	(77,7)	(76,5–78,9)
	Dois ou mais	12.147	(20,4)	(20,1–20,8)	6.322	(22,6)	(22,1–23,1)	860	(17,7)	(16,7–18,8)
	Ignorado	3.790	(6,4)	(6,2–6,6)	641	(2,3)	(2,1–2,5)	220	(4,6)	(4–5,2)
Vínculo com o provável autor da agressão†										
	Cônjuge/namorado	25.557	(42,4)	(42–42,7)	14.181	(50,0)	(49,5–50,6)	905	(18,6)	(17,5–19,7)
	Ex-cônjuge/ex-namorado	5.241	(8,7)	(8,5–8,9)	3.629	(12,8)	(12,4–13,2)	339	(7,0)	(6,3–7,7)
	Familiar	4.953	(8,2)	(8,0–8,4)	2.584	(9,1)	(8,8–9,5)	330	(6,8)	(6,1–7,5)
	Amigos/conhecidos	6.786	(11,3)	(11,0–11,5)	2.923	(10,3)	(10–10,7)	1.124	(23,1)	(21,9–24,3)
	Desconhecido	3.662	(6,1)	(5,9–6,3)	1.510	(5,3)	(5,1–5,6)	1.602	(32,9)	(31,6–34,3)
	Outros	544	(0,9)	(0,8–1,0)	334	(1,2)	(1,1–1,3)	75	(1,5)	(1,2–1,9)
Local de encaminhamento†										
	Rede da saúde	32.588	(56,0)	(55,6–56,4)	14.040	(51,1)	(50,5–51,7)	2.772	(59,7)	(58,2–61,1)
	Rede da assistência social	6.131	(13,8)	(13,5–14,2)	4.144	(19,9)	(19,4–20,5)	839	(24,5)	(23,1–26)
	Outras delegacias	15.239	(34,4)	(33,9–34,8)	7.925	(38,1)	(37,4–38,8)	1.122	(32,8)	(31,3–34,4)
	Delegacia de Atendimento à Mulher	4.486	(10,1)	(9,8–10,4)	3.075	(14,8)	(14,3–15,3)	777	(22,7)	(21,4–24,2)
	Rede de Atendimento à Mulher	1.749	(3,9)	(3,8–4,1)	1.474	(7,1)	(6,8–7,5)	491	(14,4)	(13,2–15,6)
	Ministério público	383	(0,9)	(0,8–1,0)	307	(1,5)	(1,3–1,7)	76	(2,2)	(1,8–2,8)
	Centro de referência dos direitos humanos‡	98	(0,2)	(0,2–0,3)	61	(0,3)	(0,2–0,4)	18	(0,5)	(0,3–0,8)
	Defensoria pública‡	313	(0,7)	(0,6–0,8)	332	(1,6)	(1,4–1,8)	61	(1,8)	(1,4–2,3)

Fonte: Sistema de Informação de Agravos de Notificação (SINAN).

* Teste χ^2^: todas as variáveis foram estatisticamente significativas (p<0,05);

† não totaliza 100%, pois se trata de variável com múltiplas respostas;

‡ dados incluídos nas fichas de notificação somente a partir de 2015. n: número absoluto; IC95%: intervalo de confiança de 95%.

Na Tabela 3, observa-se que em todas as UFs houve crescimento ao longo do período. Levando em consideração 2020, primeiro ano da pandemia de COVID-19 e as restrições sociais, observa-se pequena redução nas taxas em 17 estados, como Amapá, Tocantins, maioria dos estados do Nordeste, todos da Região Sul e Goiás. Essa redução refletiu na taxa do país, que passou de 162,8, em 2019, para 142,6, em 2020.

**Tabela 3:** Taxa anual das notificações de violência contra mulheres que vivem em contextos rurais no Sistema de Informação de Agravos de Notificação, segundo país, região, unidades federativas e Distrito Federal. Brasil, 2011–2020.

País, regiões, UFs e DF		2011	2012	2013	2014	2015	2016	2017	2018	2019	2020	Taxa média
Brasil		36,7	56,2	76,1	90,4	105,2	101,0	123,8	143,3	162,8	142,6	103,8
Norte		12,1	23,1	35,6	42,6	54,3	54,7	65,1	78,0	89,4	93,7	54,9
	Rondônia	9,4	15,2	15,4	41,8	33,5	44,0	61,0	86,7	71,4	90,9	46,9
	Acre	31,3	75,7	64,4	73,0	99,3	125,6	169,5	176,0	127,5	178,1	112,0
	Amazonas	14,8	42,0	82,7	75,0	121,5	128,0	131,4	157,2	200,9	209,3	116,3
	Roraima	49,1	104,5	127,4	149,1	117,1	172,9	263,9	263,8	277,4	297,9	182,3
	Pará	5,3	8,8	17,2	19,7	28,4	19,5	23,8	30,4	41,8	43,2	23,8
	Amapá	13,6	6,6	51,2	149,5	115,1	82,6	80,5	123,4	186,1	122,9	93,1
	Tocantins	41,2	55,2	62,1	85,9	91,3	117,7	144,2	170,5	167,4	153,3	108,9
Nordeste		23,7	30,4	44,8	49,8	56,9	51,7	66,9	83,6	100,3	93,0	60,1
	Maranhão	5,0	12,8	27,5	25,7	22,8	16,5	23,4	30,4	38,6	37,4	24,0
	Piauí	21,5	30,6	49,1	41,4	52,6	68,5	83,9	75,8	89,1	63,3	57,6
	Ceará	12,1	22,6	26,3	28,4	39,7	41,6	61,3	87,6	113,7	111,0	54,4
	Rio Grande do Norte	18,4	28,6	38,4	45,9	44,3	41,1	58,5	81,4	111,4	87,6	55,6
	Paraíba	9,4	15,9	17,2	20,0	37,2	28,6	38,0	48,6	63,3	65,7	34,4
	Pernambuco	64,6	74,0	104,0	116,9	117,5	101,3	144,9	171,7	203,7	173,3	127,2
	Alagoas	95,7	91,6	121,0	124,1	156,7	123,8	154,6	219,2	204,9	192,5	148,4
	Sergipe	5,5	9,5	30,1	47,5	57,8	44,9	36,1	56,1	91,1	82,6	46,1
	Bahia	15,2	19,6	31,6	41,1	47,9	45,9	52,7	63,5	76,1	80,9	47,4
Sudeste		67,6	117,8	163,2	214,9	261,5	256,1	297,2	336,4	357,3	284,2	235,6
	Minas Gerais	66,3	118,1	213,4	286,5	351,6	321,6	332,0	359,8	368,0	254,4	267,2
	Espírito Santo	20,2	37,4	71,1	94,5	143,2	168,7	201,9	277,3	317,4	301,1	163,3
	Rio de Janeiro	80,7	121,0	125,6	139,0	124,1	128,2	193,1	212,1	241,7	239,8	160,5
	São Paulo	83,3	148,6	121,0	155,1	184,5	211,0	318,9	381,6	428,8	409,7	244,3
Sul		60,4	94,8	103,5	115,2	128,1	134,0	180,0	204,2	250,7	213,9	148,5
	Paraná	35,6	64,3	99,7	106,1	117,6	138,8	183,1	189,5	213,1	211,3	135,9
	Santa Catarina	67,2	86,1	86,4	91,3	96,6	98,4	141,5	152,1	187,4	143,9	115,1
	Rio Grande do Sul	80,2	130,4	118,6	140,0	159,3	153,1	202,9	254,2	331,7	264,4	183,5
Centro-Oeste		33,1	42,6	80,1	86,3	92,0	80,4	106,5	119,1	141,4	155,1	93,7
	Mato Grosso do Sul	99,7	106,5	226,3	249,1	229,0	177,8	267,4	305,9	367,6	382,5	241,2
	Mato Grosso	11,1	15,9	24,7	44,8	49,7	42,0	50,2	51,1	48,1	74,2	41,2
	Goiás	10,7	20,2	35,5	28,0	48,4	48,7	56,6	58,3	79,5	77,5	46,3
	Distrito Federal	60,7	109,9	151,7	90,7	101,4	150,2	148,4	205,3	235,0	261,3	151,5

Fonte: Sistema de Informação de Agravos de Notificação e progressões populacionais, partindo dos censos demográficos de 2000 e 2010, do IBGE, de mulheres da zona rural, de 18 a 59 anos de idade.

UFs: unidades federativas; DF: Distrito Federal.

Na VPA, as taxas de notificação de violência contra as mulheres rurais no Brasil, conforme o modelo de regressão, tiveram dois pontos de inflexão, e as tendências foram classificadas como crescentes. As regiões Norte e Centro-Oeste tiveram dois pontos de inflexão (2011–2013 e 2013–2020), classificados como crescentes; a Sudeste teve um ponto de inflexão crescente nos primeiros quatro anos e depois estacionária; Sul e Nordeste tiveram um ponto de inflexão demostrando crescimento contínuo em todo o período. Em relação às UFs (18) e o DF tiveram um e dois pontos de inflexão com tendência crescente, cinco com tendência crescente nos três/quatro primeiros anos e depois estacionária no restante do período. Pará foi o único estado com tendência estacionária, de 2011 a 2013, e crescente no restante do período; e Goiás foi o único que teve apenas tendência estacionária em três pontos de inflexão. Na VMP, todas as taxas de país, regiões, UFs e DF apresentaram comportamento temporal crescente estatisticamente significativo (Tabela 4).

**Tabela 4:** Tendência temporal com a variação percentual anual e a variação média no período, pelo *joinpoint regression,* das taxas de notificações de violência contra as mulheres que vivem em contextos rurais no Sistema de Informação de Agravos de Notificação, segundo país, região e unidades federativas e Distrito Federal. Brasil, 2011–2020.

País, regiões, UFs e DF		Período	VPA (IC95%)	Classificação	VPM (IC95%)	Classificação
Brasil		2011–2013	**47,7** * (6,1–105,7)	Crescente	**17,9** * (10,8–25,4)	Crescente
		2013–2020	**10,5** * (5,7–15,5)	Crescente		
Norte		2011–2013	**74,2** * (43,2–112, 1)	Crescente	**25,9** * (21,3–30,6)	Crescente
		2013–2020	**14,7** * (11,7–17,8)	Crescente		
	Rondônia	2011–2020	**28,4** * (20,2–37,2)	Crescente	**28,4** * (20,2–37,2)	Crescente
	Acre	2011–2020	**17,8** * (10,3–25,9)	Crescente	**17,8** * (10,3–25,9)	Crescente
	Amazonas	2011–2013	**125,0** * (46,5–245,6)	Crescente	**33,4** * (23,1–44,6)	Crescente
		2013–2020	**14,9** * (8,5–21,7)	Crescente		
	Roraima	2011–2020	**19,1** * (12,4–26,1)	Crescente	**19,1** * (12,4–26,1)	Crescente
	Pará	2011–2013	80,8 (-9,3–260,6)	Estacionária	**26,1** * (10,8–43,5)	Crescente
		2013–2020	**13,7** * (3,7–24,7)	Crescente		
	Amapá	2011–2020	**31,7** * (8,8–59,4)	Crescente	**31,7** * (8,8–59,4)	Crescente
	Tocantins	2011–2018	**22,1** * (18,8–25,5)	Crescente	**15,2** * (10,9–19,7)	Crescente
		2018–2020	-5,9 (-23,3–15,5)	Estacionária		
Nordeste		2011–2020	**16,1** * (12,2–20,0)	Crescente	**16,1** * (12,2–20,0)	Crescente
	Maranhão	2011–2013	**138,4** * (21,5–368,0)	Crescente	**26,5** * (11,0–44,1)	Crescente
		2013–2016	-13,8 (-56,1–69,3)	Estacionária		
		2016–2020	22,8 (-0,8–52,0)	Estacionária		
	Piauí	2011–2020	**14,1** * (7,4–21,2)	Crescente	**14,1** * (7,4–21,2)	Crescente
	Ceará	2011–2013	37,0 (-11,7–112,7)	Crescente	**27,9** * (17,8–38,9)	Crescente
		2013–2020	**25,5** * (18,3–33,1)	Crescente		
	Rio Grande do Norte	2011–2020	**18,5** * (12,9–24,3)	Crescente	**18,5** * (12,9–24,3)	Crescente
	Paraíba	2011–2020	**22,9** * (17,9–28,1)	Crescente	**22,9** * (17,9–28,1)	Crescente
	Pernambuco	2011–2020	**12,2** * (8,3–16,2)	Crescente	**12,2** * (8,3–16,2)	Crescente
	Alagoas	2011–2020	**9,7** * (6,2–13,4)	Crescente	**9,7** * (6,2–13,4)	Crescente
	Sergipe	2011–2014	**101,7** * (19,7–239,7)	Crescente	**33,6** * (13,9–56,8)	Crescente
		2014–2020	8,7 (-8,8–29,7)	Estacionária		
	Bahia	2011–2014	**39,3** * (21,1–60,1)	Crescente	**20,4** * (15,4–25,7)	Crescente
		2014–2020	**12,0** * (6,9–17,4)	Crescente		
Sudeste		2011–2014	**49,2** * (21,3–83,5)	Crescente	**18,7** * (11,4–26,5)	Crescente
		2014–2020	5,9 (-1,2–13,6)	Estacionária		
	Minas Gerais	2011–2014	**69,9** * (36,3–111,7)	Crescente	**18,4** * (10,7–26,7)	Crescente
		2014–2020	-1,1 (-8,2–6,5)	Estacionária		
	Espírito Santo	2011–2013	**92,2** * (8,8–239,6)	Crescente	**35,7** * (23,6–49,0)	Crescente
		2013–2018	**31,1** * (9,5–56,9)	Crescente		
		2018–2020	4,4 (-40,9–84,5)	Estacionária		
	Rio de Janeiro	2011–2020	**11,7** * (8,0–15,6)	Crescente	**11,7** * (8,0–15,6)	Crescente
	São Paulo	2011–2020	**19,8** * (15,3–24,5)	Crescente	**19,8** * (15,3–24,5)	Crescente
Sul		2011–2020	**14,9** * (11,4–18,5)	Crescente	**14,9** * (11,4–18,5)	Crescente
	Paraná	2011–2013	**64,1** * (20,7–123,0)	Crescente	**22,7** * (15,9–30,0)	Crescente
		2013–2020	**12,9** * (8,4–17,7)	Crescente		
	Santa Catarina	2011–2020	**10,5** * (7,0–14,1)	Crescente	**10,5** * (7,0–14,1)	Crescente
	Rio Grande do Sul	2011–2020	**14,4** * (10,3–18,6)	Crescente	**14,4** * (10,3–18,6)	Crescente
Centro-Oeste		2011–2013	**52,4** * (5,0–121,2)	Crescente	**18,9** * (10,9–27,4)	Crescente
		2013–2020	**10,7** * (5,3–16,4)	Crescente		
	Mato Grosso do Sul	2011–2020	**14,4** * (7,9–21,4)	Crescente	**14,4** * (7,9–21,4)	Crescente
	Mato Grosso	2011–2014	**57,7** * (22,2–103,5)	Crescente	**21,5** * (12,4–31,4)	Crescente
		2014–2020	6,7 (-2,1–16,3)	Estacionária		
	Goiás	2011–2013	70,1 (-44,4–420,6)	Estacionária	**24,7** * (4,1–49,5)	Crescente
		2013–2017	14,8 (-34,4–100,9)	Estacionária		
		2017–2020	13,3 (-35,2–98,2)	Estacionária		
	Distrito Federal	2011–2020	**14,2** * (7,7–21,0)	Crescente	**14,2** * (7,7–21,0)	Crescente

Fonte: Sistema de Informação de Agravos de Notificação.

UFs: unidades federativas; DF: Distrito Federal; VPA: variação percentual anual; IC95%: intervalo de confiança de 95%; VMP: variação média no período.

* p<0,05. Negrito indica resultados estatisticamente significativos.

## DISCUSSÃO

Entre as características das notificações de violência física, psicológica/moral e sexual violência contra mulheres rurais, observa-se maior frequência entre mulheres de 18 a 39 anos de idade, pardas/pretas, casadas e solteiras e baixa escolaridade. A residência foi apontada como principal local de ocorrência de violência, esta foi recorrente em especial na física, psicológica/moral, sendo majoritariamente perpetrada por uma única pessoa do sexo masculino. O agressor, em sua maioria, foi o cônjuge/namorado, mas na sexual também foram apontados autores conhecidos e desconhecidos. As taxas de notificação de violência contra mulheres rurais, em sua maioria, demostraram-se crescentes ao longo do período estudado e em 2020, primeiro ano da pandemia de COVID-19, não demostrou diminuição significativa.

As características das mulheres apontadas neste estudo corroboram outras pesquisas^1,14^. As mortes violentas intencionais (MVI) vitimizam mais as mulheres jovens, e o feminicídio ocorreu em praticamente todas as faixas etárias, porém com maior prevalência das mortes ao longo da vida reprodutiva das mulheres. Entre as maiores vítimas de feminicídio e MVI, estão as mulheres negras. Além disso, nos feminicídios, o principal autor é o companheiro ou ex-companheiro da vítima, e a residência, o principal local de ocorrência^12^. O feminicídio é expressão máxima da violência de gênero, e estudos enfatizam que mulheres com notificação prévia de violência têm mais risco de feminicídio, em comparação com a população feminina em geral^14,15^.

Quanto à baixa escolaridade, em se tratando das mulheres rurais, segundo o Censo Agropecuário (2017), mais de 60,0% delas tinham o ensino fundamental. O abandono escolar das mulheres rurais é motivado, muitas vezes, pela necessidade de auxiliar no trabalho familiar, por más condições/ausência de transporte escolar e casamento precoce. Este último, no Brasil, é mais acentuado no meio rural^15^. O casamento precoce, frequentemente, tem motivação na busca da jovem por mais liberdade para sair de casa, ter uma vida melhor, sem violência ou pobreza. Mas o que acontece é que a adolescente engravida e abandona a escola, reduzindo as chances de trabalho formal, tornando-se dependente do companheiro, limitada aos serviços domésticos e vulnerável à violência^16^. O dado é reforçado pelo IBGE, que identificou, em 2023, a necessidade de trabalho, afazeres domésticos/cuidado de pessoas e gravidez como as principais razões do abandono escolar por mulheres de 15 a 29 anos, especialmente entre as mulheres pretas e pardas^17^.

A violência física também foi a mais mencionada em outros estudos com mulheres rurais^18,19^, o que pode ser pelo fato de essa violência ser mais (re)conhecida e gerar lesões visíveis e que necessitam de atendimento nos serviços de saúde^20^. Um estudo com trabalhadoras rurais em Pernambuco, sobre a percepção acerca da violência, evidenciou que grande parte dessas mulheres consideravam como violência apenas os eventos que deixavam marcas corporais^19^. Já a violência psicológica pode estar mais presente na vida das mulheres, porém pode estar subnotificada em razão de ser uma violência de difícil identificação pela própria mulher ou pelos profissionais de saúde^20,21^.

Em relação à violência sexual, os resultados chamam atenção para mulheres jovens, solteiras e pretas/pardas, tendo como agressores pessoas desconhecidas, conhecidas ou o companheiro, do sexo masculino, e acontecendo, frequentemente, uma vez. Ainda existe na sociedade a naturalização da violência sexual em determinadas situações, seja pelo parceiro acreditar que tem "direitos conjugais" sobre a mulher, seja por homens desconhecidos, ou mesmo conhecidos, estarem apoiados numa cultura patriarcal e machista de controle e imposição de poder sobre os corpos femininos, revelando a falta de segurança para as mulheres dentro e fora de casa^21^. Ademais, mulheres negras têm tido suas vidas marcadas por diversas violências ao longo dos séculos. A perspectiva interseccional contribui para a discussão da violência que perpassa as dimensões de gênero, raça e classe, que potencializam as vulnerabilidades e as desigualdades experimentadas pelas mulheres negras^22,23^.

Os resultados ainda podem estar subestimados, pois as mulheres têm dificuldades de procurar os serviços de ajuda por medo de julgamento, sentimento de culpa ou vergonha. Quando a violência sexual ocorre entre parceiros íntimos, a mulher também tende a ficar em silêncio em virtude de crenças culturais, religiosas e sociais, que mantêm as questões de relação íntimas apenas no domínio privado^24^.

As violências contra as mulheres rurais demonstraram que, majoritariamente, foram recorrentes e aconteciam na própria residência. A violência, muitas vezes, não começa com uma agressão grave, mas mascarada de violências sutis, como "excesso de proteção", "ciúmes", que fazem com que a mulher se sinta culpada e comece a tolerar as agressões, o que abre espaço para outras formas de violência, a física, patrimonial, sexual e sua repetição^25,26^. A residência, nesse caso, se torna um lugar de afetos e tensões no qual a mulher se isola por medo ou vergonha, se sente sozinha e ao longo do tempo pode desenvolver baixa autoestima, ansiedade, dependência emocional, depressão etc.^27^.

A rede da saúde foi o principal local de encaminhamento. Isso pode ocorrer por ela ser usualmente acionada nas situações mais graves de violência física ou sexual, ou quando a saúde mental já está muito afetada, então se fazem necessários outros encaminhamentos para tratamento ou acompanhamento^4,28^. Os outros locais foram as delegacias e a rede da assistência social que, geralmente, existem inclusive nos municípios de pequeno porte. Já as delegacias da mulher e a Rede de Atendimento à Mulher costumam se concentrar em cidades maiores. Segundo a Revista AzMina^29^, até 2020, apenas 7,0% das cidades brasileiras contavam com Delegacia da Mulher. Nos municípios pequenos, somente nove das 3,6 mil cidades com até 20 mil habitantes tinham Delegacia da Mulher. Ou seja, as mulheres acabam procurando delegacias comuns, nas quais nem sempre são bem acolhidas, sofrendo outra violência: a institucional^22^.

Com relação ao crescimento das taxas anuais de notificação das violências nas UFs, uma explicação para esse cenário pode ser pela melhoria na vigilância epidemiológica ao longo dos anos. Desde a universalização da notificação de violência interpessoal/autoprovocada, em 2011, verificou-se aumento substancial da cobertura do SINAN no Brasil. Por exemplo, o país passou de 2.114 municípios notificantes (38,0%), em 2011, para 4.381 municípios notificantes (78,7%), em 2018. As taxas mais elevadas no Sudeste estão relacionadas ao melhor funcionamento da vigilância (cobertura de 89,4%, em 2018)^30^.

Outra questão a ser destacada é a diminuição das taxas de notificação durante o primeiro ano de pandemia de COVID-19, possivelmente influenciada pela imposição do isolamento social. Por outro lado, essa diminuição não foi estatisticamente significativa, o que talvez indique a gravidade dos casos de violência que levaram essas mulheres, em meio a uma pandemia, a sair de casa e chegar ao setor saúde.

Apesar do aumento da cobertura de notificação, ainda existe subnotificação. Dificuldades em reconhecer os casos de violência, falta de capacitação dos profissionais para reconhecer as violências e notificar são algumas das limitações existentes^15,31–33^. Ademais, o setor saúde ainda não é totalmente reconhecido como porta de entrada para a Rede de Atendimento à Mulher em situação de violência. Quando as mulheres buscam algum apoio, geralmente, recorrem primeiro a pessoas que confiam, e quando a violência chega a extremos, como em casos de lesões físicas ou ameaças, buscam o setor saúde ou delegacias^4,7,28,34^.

Outro ponto são os municípios de pequeno porte, nos quais as mulheres podem sentir vergonha de procurar serviços de saúde para comunicar a violência sofrida, permanecendo em relações violentas durante muito tempo. Os profissionais, por sua vez, podem ter dificuldades para notificar por medo de represálias por parte do agressor^19,32^.

Para que haja ações efetivas de enfrentamento da violência, é preciso levar em conta as especificidades do contexto rural: falta de informação, ausência de telefonia e *internet*, isolamento geográfico, desprovimento de transporte público e longas distâncias até a zona urbana e entre vizinhos/familiares^4–6,35^. São necessárias intervenções no campo da saúde, como mais capacitação dos profissionais de saúde para o processo de identificação, notificação e acolhimento das mulheres rurais em situação de violência, em especial dos(as) agentes comunitários de saúde, que estão mais próximos das mulheres; integração dos serviços de saúde, assistência e segurança, pois a questão da violência requer atenção intersetorial e abrangente, que esteja inclusive presente nas áreas rurais.

As limitações deste estudo são inerentes ao uso de dados secundários, sendo que alguns registros podem apresentar baixa completude e subnotificação. Vasconcelos et al.^34^ estimaram que, em 2019, a subnotificação de violência contra mulheres no Brasil foi de 98,5, 75,9 e 89,4% para as violências psicológica, física e sexual, respectivamente. Uma mesma mulher pode aparecer mais de uma vez no banco, mas, como este não é identificado, as repetições não puderam ser excluídas.

Os resultados apresentados neste artigo ajudam a identificar as principais características das mulheres e dos eventos de violência. Os casos notificados de violência contra as mulheres rurais foram crescentes no período de 2011 a 2020 em todo o país. Ademais, destaca-se a maior vulnerabilidade das mulheres jovens, negras, casadas ou solteiras e de baixa escolaridade. Enfatiza-se que as ações de enfrentamento precisam levar em conta que as violências ocorrem, principalmente, por pessoas próximas das relações das mulheres e no próprio lar, o que torna mais difícil para a própria pessoa falar e os profissionais de saúde identificarem a violência. Incentiva-se o reforço na capacitação dos profissionais de saúde para melhorar e ampliar o processo de notificação como instrumento de cuidado com as mulheres em situação de violência, considerando os contextos rurais e suas especificidades.
